# Molecular insights into the interactions between PEG carriers and drug molecules from *Celastrus hindsii*: a multi-scale simulation study

**DOI:** 10.1038/s41598-024-67720-4

**Published:** 2024-07-22

**Authors:** Thi H. Ho, Hien Duy Tong, Thuat T. Trinh

**Affiliations:** 1 Laboratory for Computational Physics, Institute for Computational Science and Artificial Intelligence, Van Lang University, Ho Chi Minh City, 70000 Vietnam; 2 Faculty of Mechanical - Electrical and Computer Engineering, School of Technology, Van Lang University, Ho Chi Minh City, 70000 Vietnam; 3 Faculty of Engineering, Vietnamese-German University (VGU), Thu Dau Mot City, Binh Duong Province 75000 Vietnam; 4 Porelab, Department of Chemistry, Norwegian University of Science and Technology, NTNU, 7491 Trondheim, Norway

**Keywords:** Molecular dynamics, Quantum chemistry

## Abstract

Efficient drug delivery is crucial for the creation of effective pharmaceutical treatments, and polyethylene glycol (PEG) carriers have been emerged as promising candidates for this purpose due to their bio-compatibility, enhancement of drug solubility, and stability. In this study, we utilized molecular simulations to examine the interactions between PEG carriers and selected drug molecules extracted from *Celastrus hindsii*: Hindsiilactone A, Hindsiiquinoflavan B, Maytenfolone A, and Celasdin B. The simulations provided detailed insights into the binding affinity, stability, and structural properties of these drug molecules when complexed with PEG carriers. A multi-scale approach combining density functional theory (DFT), extended tight-binding (xTB), and molecular dynamics (MD) simulations was conducted to investigate both unbound and bound states of PEG/drug systems. The results from DFT and xTB calculations revealed that the unbound complex has an unfavorable binding free energy, primarily due to negative contributions of delta solvation free energy and entropy. The MD simulations provided more detailed insights into the interactions between PEG and drug molecules in water solutions. By integrating the findings from the multi-scale simulations, a comprehensive picture of the unbound and bound states of PEG and drug systems were obtained. This information is valuable for understanding the molecular mechanisms governing the binding of drugs in PEG-based delivery platforms, and it contributes to the rational design and optimization of these systems.

## Introduction

Polyethylene glycol (PEG) is a widely-used polymer, applied in multiple scientific disciplines such as drug delivery systems due to its versatility^1,2^. PEGylation, the covalent attachment of PEG chains to macro molecules or nanoparticles, enhances solubility, stability, and bio compatibility, thereby improving pharmacokinetics and pharmacodynamics profiles, leading to increased efficacy and safety of therapeutic agents^1^. In drug delivery systems, PEG coating offers numerous advantages including prolonged systemic circulation time, protection against enzymatic degradation, enhanced cellular uptake, and improved targeted delivery^2^. PEGylation has been shown to enhance therapeutic efficacy of various types of drugs, including small molecules, peptides, proteins, and nucleic acids^3,4^.

Molecular simulations has been a powerful tool for understanding the properties and behavior various materials^5–10^ including PEG-based materials, such as polymers, nanoparticles, small molecules in-silico techniques^11–16^ and hydrogels^17–20^. For instance, Lee et al.^17^ found good agreement between simulated and experimental hydrodynamic radii of PEG and polyethylene oxide (PEO) polymers in water. Brambilla et al.^20^ investigated the interaction between amyloid-beta peptide and long-circulating polymeric nanoparticles, suggesting potential for improved Alzheimer’s disease conditions. Bunker et al.^21^ reviewed the importance of molecular dynamics simulations in obtaining mechanistic insights into liposome-based drug delivery systems structure and behavior, while Magarkar et al.^22^ investigated the molecular mechanism behind PEG’s steric shielding effect on conjugated proteins. The latter study found that PEGylated protein binding affinity depends on chain length and location, providing valuable insights for therapeutic applications. It is crucial to recognize that the PEG polymer can form both unbound (non-covalent interactions) and bound (covalent bonding) complexes with drug molecules. The behavior of these two distinct systems in water solutions can be significantly different, as demonstrated by Li et al.^23^.

*Celastrus hindsii*, a plant native to Vietnam, China, and Southeast Asia, has long been used traditionally for its medicinal properties and insecticidal qualities^24^. The leaves of this plant contain flavonoids, sesquiterpenes, diterpenes, triterpenes, alkaloids, and other compounds with antioxidant and antitumor activities^24^. Ly et al. isolated rosmarinic acid oligomers from the leaves of *Celastrus hindsii*, which exhibited antioxidative activity against methyl linoleate autooxidation and radical-initiated peroxidation of soybean in liposomes^25^.

Furthermore, various bioactive compounds have been discovered in *Celastrus hindsii*, such as celahin D, an agarofuran sesquiterpene polyol ester was isolated by Huang et al.^26^, and celahin C, another sesquiterpene ester also found in the same species^27^. Su et al. demonstrated over one hundred bioactive compounds from *Celastrus hindsii* with diverse biological activities, including anti-inflammatory, analgesic, and antitumor effects^28^. New compounds such as hindsiilactone A and hindsiiquinoflavan B^29^ and maytenfolone-A^28^ have also been discovered in *Celastrus hindsii*. Additionally, other compounds such as Celasdin-A, Celasdin-C, anti-AIDS Celasdin-B, and cytotoxic maytenfolone-A were identified by Kuo et al.^30^. Recently, Viet et al.^31^ isolated a mixture of $\alpha$-amyrin and $\beta$-amyrin with antioxidant, xanthine oxidase, and tyrosinase inhibitory potential from its leaves.

The rich phytochemical composition of *Celastrus hindsii* supports its traditional use in folk medicine and as an insecticide^24,28^. However, despite their significant pharmacological potential, the clinical applications of many bioactive compounds are often hindered by challenges related to poor solubility, low bioavailability, and limited stability^2^. To address these obstacles, effective drug delivery systems can be developed, such as those based on PEG carrier systems and PEGylation. PEGylation, the process of attaching PEG chains to therapeutic molecules, has emerged as a widely adopted strategy for enhancing the stability, solubility, and pharmacokinetics of drugs^2^. By forming a protective shield around the drug molecule, PEGylation can reduce immunogenicity, protect against enzymatic degradation, and prolong circulation times in the body. Despite these benefits, recent studies have highlighted the potential for poor binding affinity between PEG and certain drug molecules, which may compromise the performance of PEG-based drug delivery systems.

In this study, we utilized molecular simulations to examine the interactions between PEG carrier molecules and a selection of bioactive compounds derived from the plant species *Celastrus hidsii*. This particular choice of compound was motivated by their historical significance in traditional folk medicine, where they have been widely employed as therapeutic agents across various applications. The rich chemical diversity exhibited by these compounds presented an exciting opportunity to study how PEG carrier molecules might engage with and enhance the delivery of such natural products. We investigate both the unbound system, which refers to non-covalent interactions between PEG and drug molecules, and the bound system, which denotes covalent bonding between PEG and drugs. The aim was to gain insights into the molecular-level details that govern their stability and behavior. To conduct the simulations, we utilize a combination of Density Functional Theory (DFT), extended Tight-Binding (xTB) methods, and classical molecular dynamics (MD) simulations. The results of the simulations reveal important insights into the nature of the PEG-bioactive compound interactions.

## Results and discussion

### DFT calculations

The results from the DFT calculations including computation of electronic structure and binding energy are reported in this section. We also discuss the Molecular Electrostatic Potential (MEP), a widely-used concept in computational chemistry for depicting molecular polarization^32^. The MEP provides valuable insights into the distribution of electron density within a molecule, which is essential for understanding its interactions with other molecules and overall reactivity^32^. Greater polarization in a molecule refers to a higher separation between its positive and negative charges, which can significantly impact the molecular interactions and binding affinity between PEG and drug molecules. A more polarized molecule generally exhibits stronger dipole moments, leading to enhanced electrostatic interactions with other polarizable species. In the context of PEG-drug complexes, greater polarization in the drug molecule can result in a stronger attraction between the polar regions of the drug and the PEG chain, contributing to more stable complex formation.

**Figure 1:**
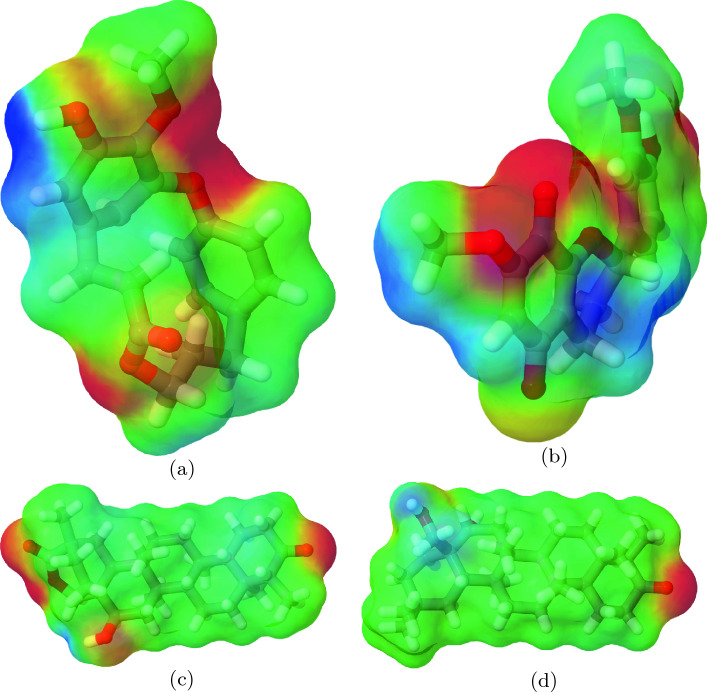
Visualization of MEP obtained from DFT calculations for drug molecules of HINA (**a**), HINB (**b**), MAYA (**c**), and CELB (**d**). The red color indicates regions of negative electrostatic potential, which are electron-rich areas. The blue color indicates regions of positive electrostatic potential, which are electron-deficient areas.

The MEP results for drug molecules, visualized in Fig. 1, indicate that HINA and HINB display greater polarization than MAYA and CELB, due to more functional groups and oxygen atoms, increasing molecular polarity. High polarization in HINA and HINB could affect interactions with PEG carriers and solvation free energy. The MEP figure for PEG.drug system (as shown in Fig. S1 of the SI) follows the same trend.

**Figure 2:**
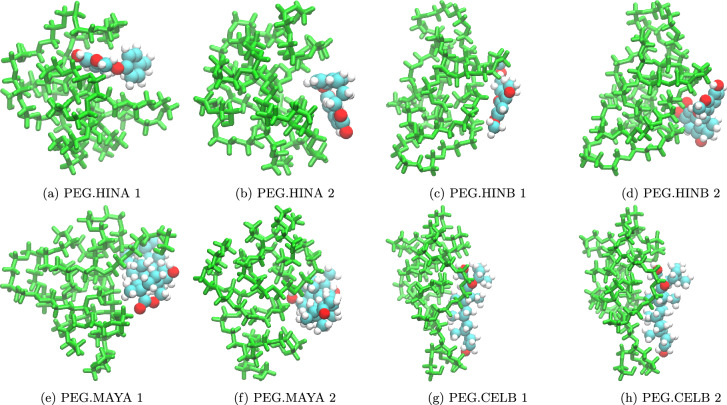
Optimized structures of unbound PEG.drug complexes obtained from DFT calculations. The PEG molecules are presented in green color for a better visualization.

As described earlier in the method sections, the optimized structures of the two lowest-energy conformers for each PEG.drug complex are presented in Fig. 2. We observed that the main interactions between PEG and drug molecules are the vdW interactions between heavy atoms. The heavy atoms primarily involved in these interactions include carbon and oxygen atoms from both the PEG and drug molecules. These interactions are particularly significant in this context because they govern the overall stability of PEG-drug complexes. These interactions contribute to the formation of stable complexes through a network of weak, non-covalent contacts that span across the PEG and drug molecules.

The DFT-computed binding energy and solvation free energy were summarized in Table 1. The values of binding energies ($\Delta$E$_{bind}^{DFT}$) reveal favorable drug-PEG interactions for all complexes as indicated by negative values between − 69 and − 171 kJ/mol. This suggests that the PEG.drug binding is energetically favorable under the gas-phase conditions.

Table 1 also presents the solvation free energies ($\Delta$G$_{solv}^{DFT}$) of the drug molecules follow the trend: HINB > MAYA > HINA > CELB. This aligns with their MEP results, where HINB shows the highest polarity and CELB displays the lowest polarity (Fig. 1). It is not surprising that PEG exhibits more favourable solvation free energies than the drug molecules due to its hydrophilic behavior^1^ , which promote stronger interactions with the water solvent.

**Table 1:** Calculated bind energy (kJ/mol) and solvation free energy (kJ/mol) obtained from DFT calculation for unbound PEG.drug complexes.

System	$\Delta$E$_{bind}^{DFT}$	$\Delta$G$_{solv, PEG.drug}^{DFT}$	$\Delta$G$_{solv, drug}^{DFT}$	$\Delta$G$_{solv, PEG}^{DFT}$	$\Delta \Delta$G$_{solv}^{DFT}$
PEG.HINA 1	− 171	− 147	− 50	− 150	54
PEG.HINA 2	− 92	− 168	− 54	− 132	17
PEG.HINB 1	− 69	− 178	− 68	− 147	37
PEG.HINB 2	− 104	− 179	− 69	− 147	37
PEG.MAYA 1	− 124	− 189	− 58	− 166	35
PEG.MAYA 2	− 107	− 179	− 55	− 162	38
PEG.CELB 1	− 163	− 160	− 44	− 163	47
PEG.CELB 2	− 159	− 166	− 43	− 169	47

Upon comparing solvation free energies of the drug molecules and their corresponding unbound complexes with PEG carriers, we observed a significant increase in solvation free energy upon complex formation (almost three times higher for the PEG.drug complexes compared to individual drug molecules). However, determining the thermodynamics of these interactions requires considering delta solvation free energy ($\Delta \Delta$G$_{solv}^{DFT}$), which ranged from 17 to 54 kJ/mol (see Table 1). The positive values of the delta solvation free energy indicate that the solvation process is not favorable for complex formation process.

**Figure 3:**
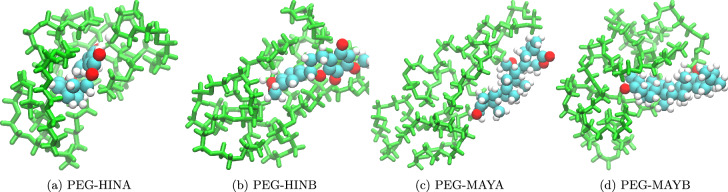
Optimized structures of covalent bound PEG-drug molecules obtained from DFT calculations. The PEG fragments are presented in green color for a better visualization.

The reaction energies of the PEGylation process for the four bound PEG-drug systems, obtained through DFT calculations, are presented in Table 2. The results reveal that the formation of PEG-HINA exhibits the most favorable $\Delta$E$_{reaction}^{DFT}$ (− 75 kJ/mol), while the formation of PEG-MAYA is unfavorable (80 kJ/mol). As demonstrated in Fig. 3, the MAYA molecule forms a specific bonding position with the PEG fragment, it generally tends to have fewer contacts with the PEG chain compared to the other drug molecules. This reduced interaction may contribute to the less favorable reaction energy observed for the formation of PEG-MAYA.

The results indicate that the PEGylation reaction leading to the formation of PEG-drug is favorable for HINA, HINB, and CELB systems, while it is unfavorable for MAYA. The interaction between the drug molecule and the PEG fragment appears to be a crucial factor in determining this reaction.

**Table 2:** Calculated reaction energy (kJ/mol) for the PEGylation process to form covalent bonded PEG-drug molecules.

System	$\Delta$E$_{reaction}^{DFT}$	$\Delta$E$_{reaction}^{xTB}$
PEG-HINA	− 75	− 31
PEG-HINB	− 40	− 5
PEG-MAYA	80	29
PEG-CELB	− 10	13

### xTB calculations

In this section, the xTB method was utilized to compute solvation free energies and binding energies of PEG.drug complexes. The xTB results indicate that drug molecules display significantly higher solvation free energies upon forming complexes with PEG carriers, consistent with the DFT findings. The solvation free energy of the complexes is approximately five times greater than that of the individual drug molecule (see Table 3). This enhancement primarily comes from PEG’s favorable solvation. The difference in solvation free energy of the complexed system ($\Delta \Delta$G$_{solv}^{xTB}$) remains unfavorable (Table 4). The positive $\Delta \Delta$G$_{solv}^{xTB}$ values, ranging from 6 to 34 kJ/mol, again imply an unfavorable solvation energy for the unbound PEG.drug systems.

**Table 3:** Solvation free energy (kJ/mol) obtained from xTB calculations for the unbound PEG.drug systems.

System	$\Delta$G$_{solv, PEG.drug}^{xTB}$	$\Delta$G$_{solv, drug}^{xTB}$	$\Delta$G$_{solv, PEG}^{xTB}$
PEG.HINA 1	− 303	− 58	− 254
PEG.HINA 2	− 310	− 58	− 258
PEG.HINB 1	− 289	− 60	− 234
PEG.HINB 2	− 273	− 60	− 235
PEG.MAYA 1	− 313	− 67	− 273
PEG.MAYA 2	− 307	− 66	− 271
PEG.CELB 1	− 278	− 57	− 254
PEG.CELB 2	− 273	− 57	− 250

Table 4 presents a comprehensive analysis of the binding energies between PEG carriers and various drug molecules, as computed by the xTB method. The table lists the calculated gas-phase interaction energies, denoted as $\Delta E_{bind}^{xTB}$, which range from − 113 to − 72 kJ/mol. These negative values indicate favorable interactions between the PEG carriers and the drugs in a gaseous state, suggesting that such complexes can form spontaneously under vacuum conditions. Upon including the solvent effects, the originally determined binding energies evolved into binding enthalpies ($\Delta$H$_{bind}^{xTB}$). The resulting values, as presented in Table 4, show that the binding enthalpy ranges from − 57 to − 79 kJ/mol. This negative values indicate that the interaction between PEG carriers and drug molecules is characterized by a net release of energy, which is typically associated with favorable or exothermic binding processes.

However, upon closer examination of the entropy contributions, which were computed through harmonic frequency analysis, it becomes evident that these interactions are not entropically favorable. The entropic effects, quantified by the change in entropy ($-T\Delta S$), yield positive values for all complexes. This result indicates that the formation of PEG-drug complexes is associated with a decrease in disorder or entropy at the molecular level. The resulting values for the binding free energies (using Eq. 3), as shown in Table 4, are predominantly positive, with a range of − 8 to 18 kJ/mol. This implies that the favorable interactions observed in the gas phase are largely negated by the entropic penalty and solvation effects upon transitioning to an aqueous environment. Moreover, the positive binding free energies suggest that the formation of PEG-drug complexes is entropy-driven, with water molecules disrupting the ordering of the system upon complexation. This entropic penalty outweighs the enthalpic benefits of the molecular interaction, leading to unfavorable thermodynamics for the stability of the complex in aqueous solution. Consequently, these findings indicate that PEG-drug complexes may be inherently unstable when exposed to water, which has significant implications for their potential application and design in pharmaceutical formulations.

**Table 4:** Calculated binding energy, solvation free energy, enthalpy, entropy and binding free energy obtained from xTB calculations for unbound PEG.drug complexes.

System	$\Delta$E$_{bind}^{xTB}$	$\Delta \Delta$G$_{solv}^{xTB}$	$\Delta$H$_{bind}^{xTB}$	− T$\Delta$S$^{xTB}$	$\Delta$G$_{bind}^{xTB}$
PEG.HINA 1	− 81	9	− 72	64	− 8
PEG.HINA 2	− 72	7	− 65	73	8
PEG.HINB 1	− 80	6	− 74	74	0
PEG.HINB 2	− 79	22	− 57	69	12
PEG.MAYA 1	− 85	26	− 59	76	18
PEG.MAYA 2	− 89	30	− 59	77	18
PEG.CELB 1	− 102	34	− 68	83	14
PEG.CELB 2	− 113	34	− 79	87	8

All value units are in kJ/mol.

The present results highlights the significance of accounting for both thermodynamic and entropic factors when engineering drug delivery systems based on compounds derived from *Celastrus hindsii* and PEG carriers. While the binding energy between PEG and the drug compound are favorable, the substantial unfavorable entropy change and delta solvation free energy contribute to the overall instability of the complex. The findings from static and implicit water simulations provide valuable insights; however, these approaches might be limited in their ability to accurately capture the intricate interactions between PEG and the drug carrier in a more realistic aqueous environment. To verify and further elucidate these results, a series of MD simulations were conducted. These simulations have incorporated explicit water molecules, allowing for a more detailed investigation into the dynamic behavior and interaction between PEG and the drug carrier in solutions.

### MD simulations

**Figure 4:**
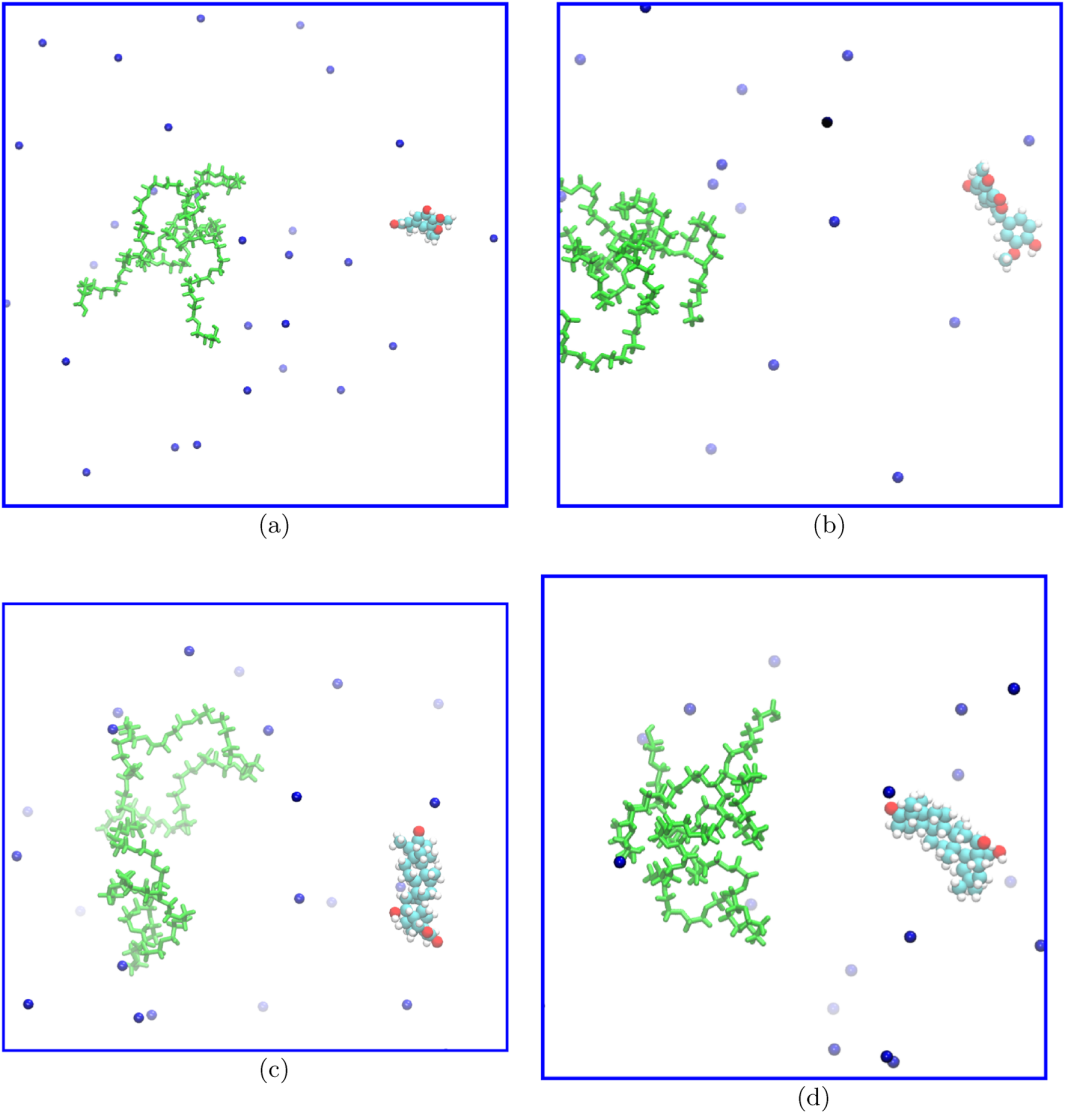
Representative snapshots of the last MD frame for the unbound systems after 100 ns of simulations, demonstrating the separation of the PEG carrier and the bioactive drug molecules. The green and blue colors represent the PEG and Na, respectively.

In the molecular dynamics study, we first analyzed the separation of PEG carrier and bioactive drug molecules during the simulation process. As depicted in Fig. 4, which shows a typical snapshot of the last MD frame for the unbound system after 100 ns of simulations, it is evident that the PEG carrier and the drug molecule have indeed separated from each other. This observation aligns with findings reported in previous literature on the interaction between PEG and small molecules in the unbound state^23^. The MD simulations, in conjunction with previous DFT and xTB simulations, consistently demonstrate the separation of the PEG carrier and bioactive drug molecules. This observation shows the reliability and consistency of the computational methods employed in studying the behavior of these systems.

**Figure 5:**
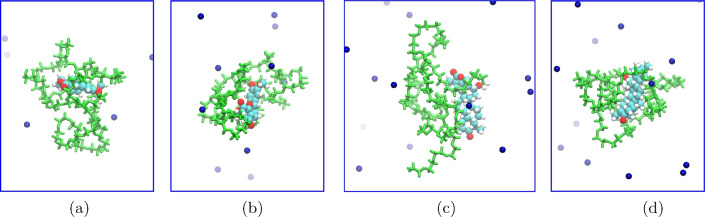
Representative snapshots of the last MD frame for the bound systems after 100 ns of simulations, demonstrating no separation between the PEG carrier and the bioactive drug molecules due to the formation of chemical bonds. The green and blue colors represent the PEG and Na, respectively.

In contrast to the unbound system, when examining the bound system PEG-drug, we observe that no separation occurs after 100 ns of molecular dynamics simulations (Fig. 5). This striking difference between the unbound and bound systems is attributed to the formation of chemical bonds between small molecules and PEG molecules. The establishment of these strong chemical interactions significantly enhances the overall interaction between the components, effectively preventing their dissociation from one another.

#### Solvent accessible surface area

**Figure 6:**
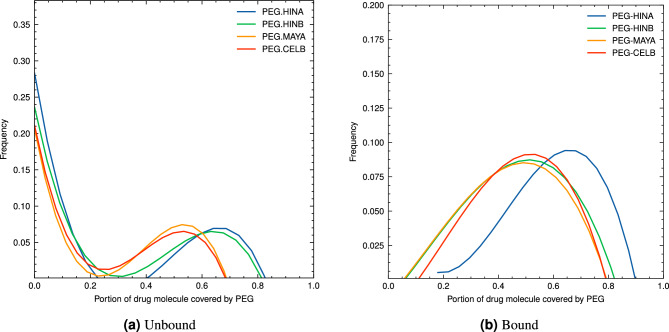
Histograms of the fraction of drug surface covered by PEG for unbound (**a**) and bound (**b**) systems.

The solvent-accessible surface area (SASA) analysis is one of the most important properties of the PEG and small drug molecules interaction^23^. We calculated the SASA for each sampled configuration of both unbound and bound systems. By determining the portion of a molecule’s surface area covered by PEG, we gained insights into their dynamic molecular mechanisms. The SASA calculations included assessing total surface areas and interactions with the PEG polymer, considered as a part of the solvent. Combining results from all sampled configurations in histograms (Fig. 6) revealed trends and patterns on how these compounds interact during the simulations.

In the unbound systems, we observed that there was generally no significant interaction between the PEG carrier and bioactive drug molecules from *Celastrus hindsii* for most of the simulation time. Consequently, the coverage by PEG over these compounds remained at very low portion (< 0.2) for a majority of the sampling configurations (See Fig. 6a). However, a distinct peak is observed for PEG coverage at a very low frequency. This indicates that during the MD simulation, the PEG carrier and drug molecule only interact in a very small fraction of simulation time. Furthermore, we found that the PEG coverage for the unbound systems involving PEG.HINA and PEG.HINB were approximately 0.65, while those with PEG.MAYA and PEG.CELB exhibited coverage values of around 0.55. This difference in PEG coverage can be attributed to the size disparities between HINA/HINB and MAYA/CELB molecules. Smaller molecules tend to have a higher coverage percentages with the PEG carrier, which may influence their overall behavior within the PEG-based drug delivery system.

In constranst, bound systems displayed a significantly different behavior compared to unbound systems, with PEG effectively covering the bioactive drug molecules from *Celastrus hindsii* most of the time. The absence of low portion peak and a wider PEG coverage range (0.1–0.9) in the histogram data confirmed that chemical bonds favored the interaction between PEG and drug molecules in solutions (See Fig. 6b). In the unbound system, the highest frequency is observed around 0.0 for PEG coverage, indicating that the unbound compounds tend to be separated. However, the bound system exhibits a peak of high frequency at approximately 0.5, suggesting that in the bound state, the compounds are more closely associated, leading to higher PEG coverage values. This observation highlights the potential benefits of PEGylation for enhanced PEG coverage and for protection of the small drug molecules.

To further investigate the conformational behaviour of the bound compounds, additional calculations on another conformer of the bound systems, PEG-drug 2, were performed (see SI). The results (Table S1) show that the xTB-calculated relative energy of the PEG-drug 2 system is higher than that of the PEG-drug system. Due to the high computational cost of the DFT method, we did not perform the DFT calculations for the PEG-drug 2 systems. The SASA analysis from the MD simulations of the PEG-drug 2 conformer (See SI) exhibits a similar pattern as observed for the PEG-drug systems. This indicates that the drug molecule in this conformation also experiences increased PEG coverage when bound to the PEG carrier.

#### Radius of gyration

**Figure 7:**
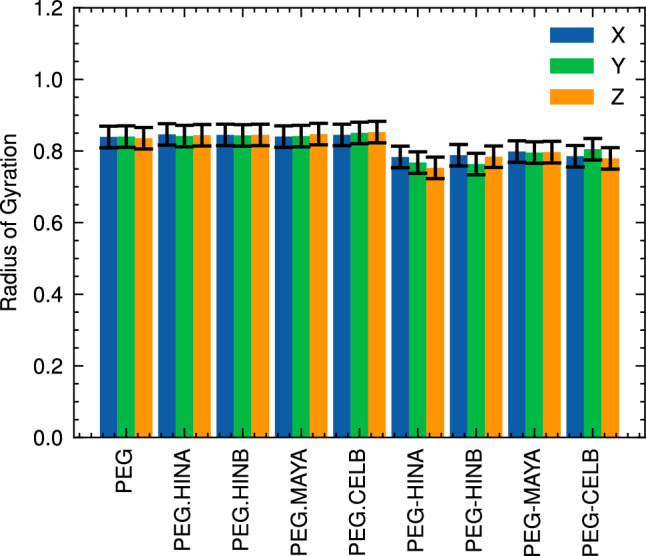
x, y, z components of the radius of gyration of PEG molecule in both unbound (PEG.drug) and bound (PEG-drug) systems.

We also calculated the radius of gyration (Rg) for PEG molecules in both unbound and bound configurations to supplement the SASA analysis. The results demonstrated that there was no significant difference observed between the Rg values for PEG in solution and those encountered within the unbound system simulations (see Fig. 7). These findings were consistent with previous molecular dynamics studies on similar PEG-based systems^23^, further validating the accuracy of the methodology. The average Rg value obtained for PEG molecules across all unbound systems was approximately 0.85 nm, which in excellent agreement with previous study^23^.

In contrast to the findings for unbound systems, we observed a reduction in the Rg values (around 0.77 nm) for PEG carrier when bound with bioactive drug molecules. This observation suggests that an attractive nonbonded interaction may be occurring between the PEG and these compounds, causing the PEG polymer reducing the Rg.

#### PEG and drug interaction

In order to gain further insights into the complex interplay between polyethylene glycool (PEG) and drug molecules during MD simulations, we focused our analysis on characterizing van der Waals (vdW) interactions. These non-covalenent forces are known to be critical in mediating molecular recognition and binding processes within many bio-macromolecular systems. To systematically quantify the vdW interactions between PEG and drug molecules, we adopted a rigorous distance-based approach. Specifically, any atomic pair with an interatomic distance of less than 3.0 Å was deemed to exhibit vdW interactions. This well-defined criterion enabled us to objectively assess the strength and distribution of vdW interactions in both unbound and bound complexes.

**Figure 8:**
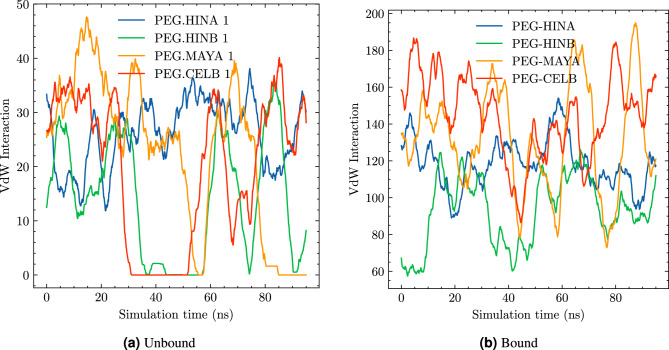
Number of vdW interaction between PEG and drug molecules in the unbound systems (**a**) and bound system (**b**).

As depicted in Fig. 8, our analysis reveals a striking disparity in the frequency and distribution of vdW interactions between the two systems. Notably, in the case of unbound PEG-drug pairs, the number of vdW interactions remains remarkably low throughout all simulated trajectories. This indicates that the complexation between PEG and drug molecules is not stable, as they tend to separate in solution.

In contrast to the unbound systems, our MD simulations revealed a significant difference in vdW interactions for the bound complexes. Specifically, we observed that the number of vdW interactions between PEG and bound drug molecules was nearly four times higher compared to the unbound systems. This substantial increase in vdW interaction frequency suggests that the presence of a chemical bonding between PEG-drug significantly enhances their binding affinity. Such an increase in binding affinity can have profound implications for the stability and efficacy of PEGylated therapeutics, particularly when exposed to physiological environments.

Our comprehensive analysis of the PEG-drug complex revealed a striking absence of hydrogen bonds between these two molecules. This finding underscores the unique chemical properties of PEG, including its hydrophilic and flexible nature, which enable it to form strong non-covalent bonds with drug molecules through vdW forces. Our MD simulations demonstrate that van der Waals interactions dominantly mediate PEG-drug recognition and binding, with a substantial increase observed upon formation of a chemical bond.

### Discussion

In this section, we will compare and contrast the performance of the xTB and DFT methods in the context of investigating PEG-drug interactions. The xTB approach is known for its computational efficiency and lower accuracy compared to DFT^33^. Despite its limitations, xTB can serve as a useful tool for providing preliminary insights into the behavior of complex systems. Here, we aim to assess the level of agreement between the results obtained from xTB and DFT calculations for PEG-drug interactions. This comparison will help us understand the reliability of xTB in predicting the binding energy and solvation free energy of these complexes.

**Figure 9:**
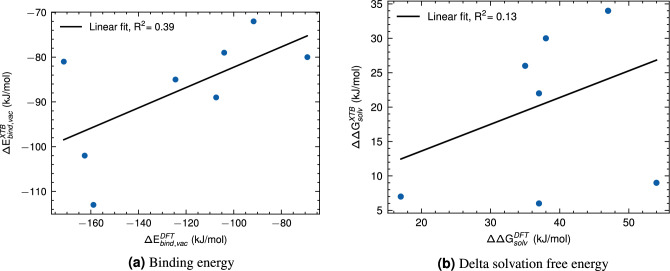
Comparison of calculated values obtained from DFT and xTB calculations for binding energy (**a**) and for delta solvation free energy (**b**).

As depicted in Fig. 9, we observed that the xTB method can capture the trend of the DFT calculations regarding the binding energy between PEG and drug molecules. However, the DFT-calculated binding energies were more negative than the corresponding xTB-calculated binding energies, which suggests a stronger binding interaction between the PEG and drug molecules in the case of DFT calculations. While it would be ideal to compare these values with experimental data, to the best of our knowledge, there are no available experimental data or other DFT data for the binding energy of these specific complexes. To advance the field and address this gap, we propose that future experimental work should focus on measuring the binding free energy for the unbound PEG.drug complex. This would not only confirm the computational trends but also provide a critical benchmark for the accuracy of both DFT and xTB methods in predicting such interactions. Furthermore, extending the scope of future experimental studies to include the energies associated with the PEGylation reactions themselves would be highly beneficial.

Our MD simulations have provided valuable insights into the interplay between PEG and a variety of small drug molecules, comparable to the work established by Li et al.^23^. The consistency between our MD simulation results and those from the Li et al.^23^ study is remarkable. Both sets of simulations indicate that PEG exhibits an unfavorable interaction with small drug molecules like paclitaxel and piroxicam. This unfavorability manifests as a propensity for the drug and PEG moieties to separate over time within the simulation environment, suggesting that such small drug and PEG complexation may not be stable in physiological conditions. However, the interaction dynamics change markedly when a larger drug molecule with a porphin ring, such as hematoporphyrin. In contrast to the behavior observed with smaller drugs, MD simulations reveal a strong and stable interaction between PEG and the hematoporphyrin molecule^23^. This notable difference in interaction strength suggests that the presence of a porphin ring significantly influences the binding affinity between PEG and the drug molecule. The interaction observed with hematoporphyrin can be attributed to favorable lipophilic interactions between the nonpolar –$\hbox {CH}_{2}$– groups of the PEG and the porphin ring^23^.

The delta solvation free energy is a critical component of the overall binding free energy for PEG-drug complexes. The analysis showed that the level of agreement between the xTB and DFT methods was notably weaker when examining the delta solvation free energies, as demonstrated in Fig. 9b. The linear regression’s coefficient of determination (R$^2$) for this comparison is only 0.13, which indicates a very low correlation between these methods. This discrepancy can be attributed to the less accurate auxiliary basis method employed in xTB and the ALPB model when compared to more sophisticated solvation techniques available in VASP software for DFT-based calculations. This observation underscores the importance of considering both binding and solvation free energies in evaluating computational methods for predicting PEG-drug interactions, as well as highlights the limitations of xTB when it comes to solvation free energy calculations relative to DFT. However, it is important to note that despite its limitations, xTB remains a valuable tool for providing preliminary insights into large and complex systems due to its computational efficiency.

While both the xTB and DFT methods, when employed using static calculations, provide valuable insights into the structural properties and binding affinity of PEG.drug complexes, they do not account for the dynamic behavior of these systems at finite temperatures or their explicit interactions with water solvent molecules. This limitation can result in an incomplete picture of the forces governing the formation and stability of these complexes. The MD approach, on the other hand, offers a more dynamic perspective by simulating the system’s behavior at finite temperatures and accounting for the explicit interactions with solvent molecules. This enabled us to observe how the intricate interactions between water and PEG-drug systems influence their overall properties. For instance, we discovered that the majority of the time, there is no interaction between PEG and drug molecules in unbound configurations, which aligns well with the less favorable binding energies calculated using DFT simulations.

While the xTB method stands out among semi-empirical quantum chemical methods (such as PM6, AM1, and SM6) for its enhanced accuracy and computational efficiency, it is crucial to recognize the inherent limitations of this approach^33^. The xTB operates on a framework that incorporates empirical parameters and theoretical approximations to simulate electron correlation effects to strike a balance between predictive power and computational demand. Despite these improvements, xTB is not without its constraints; it inevitably falls short of the precision achievable through experimental observations or more sophisticated ab initio methods based on DFT. The semi-empirical nature of xTB implies that while it can reproduce many properties of molecules and materials with remarkable accuracy, particularly when compared to other methods in its class, it does not capture every nuance of the electron dynamics that would be described by more rigorous theoretical frameworks^33^. DFT, for instance, relies on accurate exchange-correlation functionals to model the electron density and typically provides results that are in closer agreement with experimental findings than semi-empirical approaches.

The MD simulations align well with DFT and xTB results, particularly when considering the separation of unbound PEG-drug complexes. The unfavorable binding free energy, as calculated by both the xTB and DFT methods, indicated a high likelihood that these complexes would remain unbound in solution. This prediction is consistent with the observed MD simulation results, which revealed that drug molecules maintained a considerable distance from PEG carrier molecules even under physiological conditions. These findings collectively support the notion that the unfavorable binding energetics contribute to the separation of these complexes, thereby limiting their formation in solution.

In our study, we observed a substantial increase in vdW interactions between PEG-drugs upon the formation of chemical bonds. This enhancement in vdW interactions contributes significantly to their overall binding affinity, ultimately leading to improved PEG coverage and SASA. This indicates that the presence of a covalent bond between PEG and drug molecules plays a crucial role in maintaining close proximity during MD simulations. This stable connection prevents the PEG and drug molecules from separating during simulation time-steps, thereby ensuring consistent vdW interactions and reinforcing their binding affinity. In contrast, when no covalent bond is present, the PEG and drug molecules are more likely to fluctuate and separate throughout the MD simulations, leading to weaker vdW interactions and reduced SASA and PEG coverage.

It is important to note that while the MD simulations offer valuable insights into the dynamic behavior of PEG-drug complexes, they also come with their own limitations, such as the need for accurate force fields and the computational cost associated with long simulations^21^. Therefore, it is essential to carefully consider the appropriate level of theory and simulation methods when studying these systems. In the context of implicit solvent modeling, Wang et al. have recently introduced a novel computational method that aims to refine the prediction of binding energies through a sophisticated approach involving a three-trajectory realization at the energy minimization endpoint^34^. The potential limitations of the current xTB approach for solvation energy calculations could also be improved by employment of an alternative method grounded in statistical mechanics calculations^35^. These approaches should be included in the future simulations studies of the interaction between PEG and drug molecules.

## Conclusions

The study utilized a multi-scale computational approach that combined DFT, xTB , and molecular dynamics simulations to examine the interaction between polyethylene glycol (PEG) carrier systems and bioactive drugs derived from *Celastrus hindsii*. By examining the binding energies, solvation free energies, and dynamic interactions in both bound and unbound states, we gained insights into these complex molecular processes.

The simulations revealed that both the DFT and xTB methods predicted unfavorable binding free energies (in the range − 8 to 18 kJ/mol) for the PEG-drug system. This can be attributed to delta solvation free energy and entropy considerations. The entropic penalty of confining the PEG.drug complexes outweighing any gains in binding energy. Unfavorable solvation free energies of the complexation process may also drive these interactions towards less stable configurations. Therefore, researchers should consider these factors in formulating effective pharmaceutical products using PEG-based nanocarriers loaded with bioactive compounds.

Our MD simulations offer compelling evidence that PEG carriers and small drug molecules infrequently interact when unbound. The results also reveals that PEGylating drug molecules significantly increases stability compared to unbound systems. This improvement can be attributed to enhanced interactions between small molecules and the PEG polymer, facilitated by chemical bond formation. Solvent-accessible surface area analyses further demonstrate that PEG coverage of drug molecules persists during specific time intervals, highlighting their potential as nanocarriers for improving drug solubility and bioavailability.

While this study marks the first attempt to explore PEG-drugs in water and investigate covalent bonding effects through MD simulations, we acknowledge certain limitations. Accurate simulation of drug release, including chemical bond breaking and forming, necessitates advanced methods such as ab initio molecular dynamics (AIMD) to surpass the constraints inherent in classical force fields.

It is important to note that while this study provides valuable insights into the interactions between PEG carrier systems and bioactive drugs derived from *Celastrus hindsii*, further experimental studies are needed to validate these findings and assess their implications for the design and optimization of PEG-based delivery platforms. Additionally, further investigation could focus on the chemical activity of PEGylated drug molecules and the kinetics of the PEGylation process, including compound association/dissociation rates. A deeper understanding of these dynamics could lead to enhanced control over drug release profiles and increased therapeutic potential of PEG-based nanocarriers loaded with the bioactive compounds.

## Models and methods

### Models

**Figure 10:**
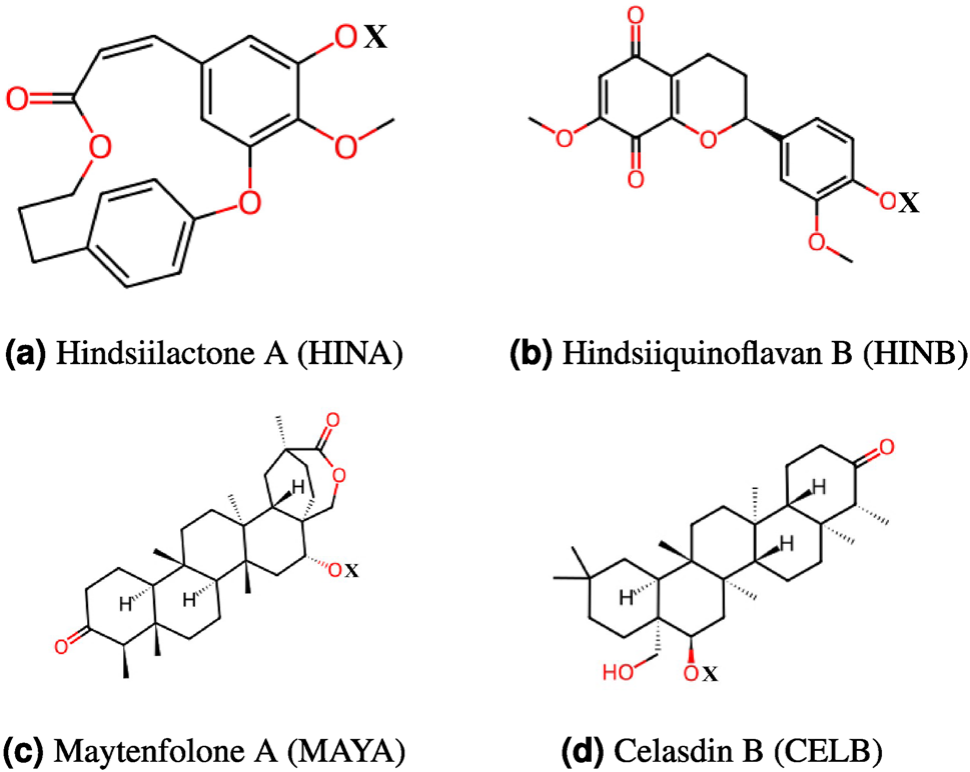
Chemical structures of selected bioactive compounds from *Celastrus hindsii* in this study. Illustration of unbound complexes (X = H) and bound PEG-drug molecule with PEG chain (X = PEG) for a better visualization of binding positions.

The chemical structures of selected bioactive compounds are presented in Fig. 10. We adopted a concise notation to represent the chemical structures of the drug molecules. For clarity and brevity throughout the manuscript, we refer to these compounds as HINA (Hindsiilactone A), HINB (Hindsiiquinoflavan B), MAYA (Maytenfolone A), and CELB (Celasdin B). The PEG molecule was the 45 monomer unit linear chain polymer, also known as PEG2000^1,2^.

We modeled both unbound and bound states between PEG and drug molecules. In the unbound state (X = H in Fig. 10), initial interactions are dominated by van der Waals (vdW) forces, with no covalent bonding (PEG and drug molecules exist as separate entities). This contrasts with bound state interactions where bonding occurs between PEG carriers and drug molecules, stabilizing the complex (X = PEG in Fig. 10).

### Methods

To understand the interactions between PEG carriers and drug molecules, we utilized DFT, xTB and MD simulations. These techniques offered insights into the binding properties, electronic structure, and overall behavior of the PEG-drug complexes, enabling investigation of their potential in drug delivery systems. By combining results from these various simulation methods, a comprehensive understanding of the interactions between PEG carriers and *Celastrus hindsii*’s bioactive compounds was achieved.

DFT calculations were conducted using the Vienna ab initio simulation package (VASP version 5.4)^36,37^ with the projected augmented wave (PAW) pseudo-potential method^38^. The generalized gradient approximation Perdew-Burke-Ernzerhof functional (PBE)^39^ was used for exchange-correlation effects, and the semi-empirical DFT-D2 dispersion correction method^40^ was employed to treat van der Waals interactions. A 400 eV cutoff energy, $\Gamma$ point Brillouin zone summation with Gaussian broadening of 0.1 eV. The selected simulation box, with a 30 Å$\,\times \,$30 Å$\,\times \,$30 Å cubic cell and a 10–15 Å vacuum layer surrounding the PEG-drug complex, ensures that self-interactions between periodic images are minimized while maintaining reasonable computational efficiency. The molecules were placed in the center, fully relaxed until forces were below 0.01 eV/Å, and single-point calculations for isolated and complex systems yielded interaction energy and charge density analysis through Bader charge analysis method^41^. We employed the implicit solvation model implemented in the VASPSol package^42,43^ to calculate the solvation free energies of PEG and drugs molecules.

The extended tight-binding (xTB version 6.0)^33,44^ method is a semi-empirical quantum chemistry approach employed in DFT simulations, striking a balance between computational efficiency and accuracy. This approach makes it particularly well-suited for investigating large systems that would be computationally costly to simulate with full DFT methods^44^. The xTB method represents a significant advancement in the field of computational chemistry, stemming from its foundation within the Density Functional Tight Binding (DFTB) framework. One of the primary strengths of xTB lies in its ability to efficiently model complex systems containing thousands of atoms, a task that would be computationally infeasible with traditional methods. The method’s efficiency is attributed to its use of pre-computed two-center integrals, which are meticulously parameterized for each atomic type across the periodic table, extending up to radon (Z = 86)^33^. This comprehensive set of parameters ensures that xTB can be applied to a wide array of chemical systems, including but not limited to organic molecules, metal-organic frameworks (MOFs), and transition-metal complexes. The parameterization of xTB is not solely about covering a broad range of elements; it also includes the sophisticated modeling of molecular geometries, vibrational frequencies, and non-covalent interactions. These features are critical for accurate simulations of chemical systems, making xTB an invaluable asset for computational chemists who require precise predictions regarding molecular properties and system behavior^44^. As a result, xTB has established itself as a robust and versatile tool that bridges the gap between empirical models and state-of-the-art ab initio calculations, offering researchers a powerful platform for exploring the vast landscape of chemical systems with confidence and precision.

In xTB simulations, we employed GFN2-xTB method^33,44^ for studying the interaction between PEG carriers and drug molecules. The xTB method was used to optimize initial system structures, calculate frequency and solvation free energy. The solvation free energy was computed using the Analytical Linearized Poisson-Boltzmann (ALPB) model, offering insights into complexes’ behavior in aqueous environments and their interaction strength affected by solvation.

To ensure reliability and convergence in our DFT and xTB calculations for each PEG-drug complex, we systematically tested multiple starting geometries and orientations. For each system, we employed several random initial geometries to sample diverse starting conditions, which were then optimized using both DFT and xTB methods. To assess convergence in total energy, we carefully compared results from these various optimization approaches. This comparative analysis confirmed consistent outcomes across different conformations and initial conditions. Consequently, our rigorous testing protocol bolsters confidence in the accuracy and robustness of the derived conclusions.

Binding energy is an essential concept in understanding the interaction between PEG and a drug molecules. It refers to the energy released when the two components combine to form a complex, which can provide insights into the strength and stability of their association. The binding energy ($\Delta E_\text {bind}$) between the PEG carrier and drug molecule can be represented by the following equation:

1 $$\begin{aligned} \Delta E_{bind} = E_{PEG.drug,opt} - (E_{PEG,opt} + E_{drug,opt}) \end{aligned}$$

where $E_\text {PEG.drug,opt}$ is the energy of the PEG.drug complex, $E_\text {PEG,opt}$ and $E_\text {drug,opt}$ are the isolated energies of the PEG carrier and drug molecule, respectively.

In addition to the binding energy, it is also important to consider the binding free energy ($\Delta G_{bind}$) of the complex formation^45^. This value takes into account not only the binding energy but also the changes in solvation free energy and entropy as the following equation:

2 $$\begin{aligned} \Delta G_{bind} = \Delta E_{bind} + \Delta \Delta G_{solv} - T \Delta S = \Delta H_{bind} - T \Delta S \end{aligned}$$

where $\Delta H_{bind}$ is the enthalpy change upon complexation, *T* is the absolute temperature, and $\Delta S$ is the entropy change. A negative value for $\Delta G_{bind}$ indicates a favorable and stable interaction between the PEG carrier and drug molecule. The term $\Delta \Delta G_{solv}$ represents the difference in solvation free energy between the PEG-drug complex and the sum of the solvation free energies of the individual components:

3 $$\begin{aligned} \Delta \Delta G_{solv} = \Delta G_{solv, PEG.drug} - (\Delta G_{solv, drug} + \Delta G_{solv, PEG}) \end{aligned}$$

The PEGylation reaction between PEG and drug to form bound molecules and water is described as:

4 $$\begin{aligned} \hbox {PEG} + \hbox {drug} = \hbox {PEG}-\hbox {drug} + \hbox {H}_{2}\hbox {O} \end{aligned}$$

The PEGylation reaction energy ($\Delta E_{react}$) was calculated as:

5 $$\begin{aligned} \Delta E_{react} = (E_{PEG-drug} + E_{water}) - (E_{PEG} + E_{drug}) \end{aligned}$$

where $E_{PEG-drug}$, $E_{water}$, $E_{PEG}$, and $E_{drug}$ are the total energies of the bound PEG-drug, water, free PEG, and free drug molecules, respectively.

The MD simulations employed the OPLS-AA force field^46^ for PEG and drug molecules, with TIP3P water models^47^. The success of the OPLS-AA force field in previous studies on PEG thermodynamics^48^ and small drug interactions^23^ supports this choice. The Desmond software^49^ version 2022.4 was used for the MD simulations. The time step was set to 2 fs and the simulations were conducted at 310 K temperature, 1 bar pressure, and under the physiological conditions (0.15 M NaCl). The Isothermal-Isobaric ensemble (NPT) was used. The Lennard-Jones interactions were cut off at 1.0 nm. After reaching equilibrium for 20 ns, each simulation ran for 100 ns, repeated across 3–5 replicates. Following completion of MD simulations for both bound and unbound systems, we analyzed PEG.drug molecular interactions by calculating the radius of gyration and the solvent-accessible surface area (SASA).

Starting with the initial 3D coordinates of the drug molecule retrieved from the PubChem database^50^, we performed a thorough geometry optimization using both xTB and DFT methods to refine its structure prior to molecular dynamics simulations. Initial PEG-drug complexes were constructed by positioning one drug molecule alongside one PEG chain for each compound (HINA, HINB, MAYA, and CELB), with an initial intermolecular distance of 5 Å. Subsequent geometry optimization was performed using the OPLS-AA force field. After complex formation, short-duration MD simulations were run for initial relaxation and conformational adjustment. For each unbound structure, we selected the two lowest-energy conformers as the initial structures. For the covalent bound PEG-drug complexes, only the lowest-energy conformer was selected. These conformers were then used for further calculations with the xTB and DFT methods. The approach of utilizing multiple short MD simulations to sample molecular interactions has been established for decades^51,52^. Alternatively, a more recent study^53^ have also proposed a new method that employ extensive conformational sampling with end point protocols to achieve a higher level of accuracy in characterizing host-guest interactions.

In the unbound systems, we selected two conformers from DFT simulations for PEG and drug complexes, namely PEG.drug 1 and PEG.drug 2. For the bound systems, only one conformer was chosen (PEG-drug) for each drug. The details of the MD simulations are presented in Table 5.

**Table 5:** Details of MD simulations used in this study including the number of NaCl (N_NaCl_), the number of water molecules (N_water_), the total number of atoms (N_atoms_), the box size (Å) and the simulation time.

System	N_NaCl_	N_water_	N_atoms_	Box size (Å)	Simulation time
PEG	30	10,776	32,706	69.409	3$\,\times \,$100 ns
PEG.HINA 1	30	10,759	32,697	69.506	5$\,\times \,$100 ns
PEG.HINB 1	30	10,754	32,679	69.398	3$\,\times \,$100 ns
PEG.MAYA 1	30	10,733	32,657	69.453	3$\,\times \,$100 ns
PEG.CELB 1	30	10,737	32,672	69.352	3$\,\times \,$100 ns
PEG.HINA 2	30	10,768	32,724	69.487	3$\,\times \,$100 ns
PEG.HINB 2	30	10,762	32,703	69.378	3$\,\times \,$100 ns
PEG.MAYA 2	30	10,747	32,699	69.585	3$\,\times \,$100 ns
PEG.CELB 2	30	10742	32687	69.341	3$\,\times \,$100 ns
PEG-HINA	30	10,755	32,682	69.353	5$\,\times \,$100 ns
PEG-HINB	30	10,756	32,682	69.483	3$\,\times \,$100 ns
PEG-MAYA	30	10,737	32,666	69.425	3$\,\times \,$100 ns
PEG-CELB	30	10,726	32,636	69.262	3$\,\times \,$100 ns

### Supplementary Information

Supplementary Information.

## Data Availability

The datasets generated and/or analyzed during the current study are available from the corresponding author on reasonable request.
