# Supplementary material for: Molecular insights into the interactions between PEG carriers and drug molecules from Celastrus hindsii: a multi-scale simulation study
Source: Sci Rep. 2024 Jul 22;14:16777. doi: 10.1038/s41598-024-67720-4 (PMC11263547; doi:10.1038/s41598-024-67720-4)
Supplement: Supplementary file 1 — Supplementary Information. [file 41598_2024_67720_MOESM1_ESM.pdf]

Supporting Information: Molecular Insights into the Interactions between PEG Carriers and Drug Molecules from Celastrus hindsii: A Multi-scale Simulation Study

Thi H. Ho,[†,‡] Hien Duy Tong,[¶] and Thuat T. Trinh[*,§]

†*Laboratory for Computational Physics, Institute for Computational Science and Artificial Intelligence, Van Lang University, Ho Chi Minh City, 70000, Vietnam*

‡*Faculty of Mechanical - Electrical and Computer Engineering, School of Technology, Van Lang University, Ho Chi Minh City, 70000, Vietnam*

¶*Faculty of Engineering, Vietnamese-German University (VGU), Thu Dau Mot City, Binh Duong Province 75000, Vietnam*

§*Porelab, Department of Chemistry, Norwegian University of Science and Technology, Høgskoleringen 5, 7491-Trondheim, Norway*

E-mail: thuat.trinh@ntnu.no

In the Supplementary Information, we present the Snapshot of the system PEG.HINA 1, Molecular Electronic potential (MEP) maps of selected PEG.drug complexes to provide a detailed understanding of their electronic properties. Additionally, we include simulations of another conformer of bound system PEG-drug 2. The optimized structure, relative energy, and drug surface coverage for these systems are also provided.

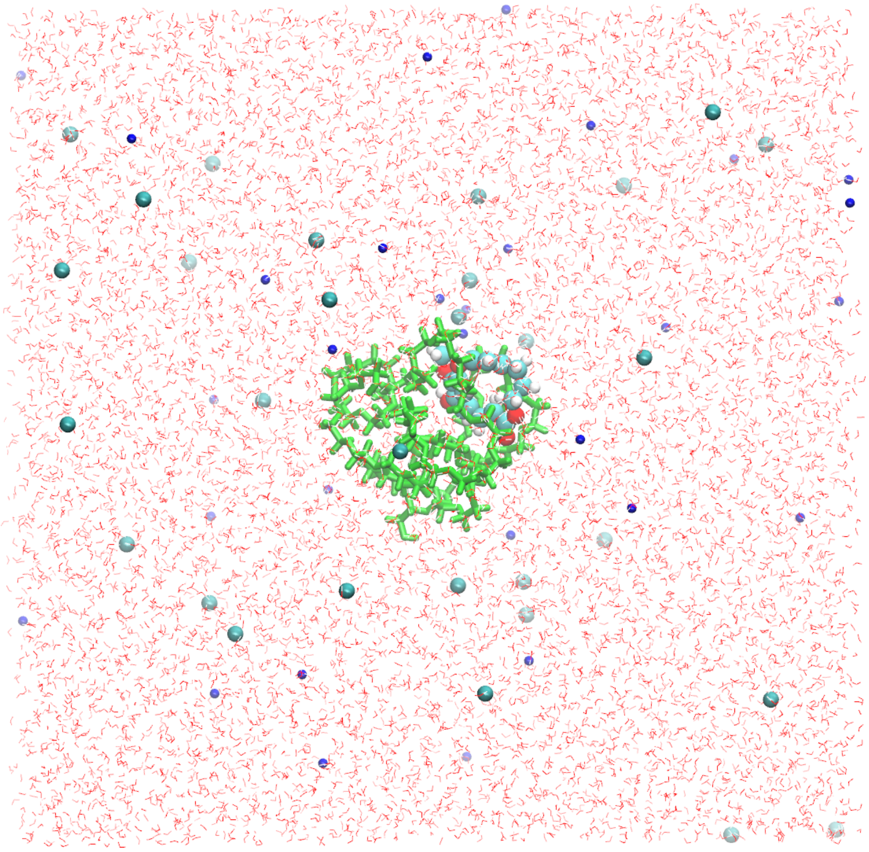

Figure S1: Snapshot of the system PEG.HINA 1 at the beginning of MD simulations. The PEG molecule depicted in green and water represented by red sticks for enhanced visual clarity.

Table S1: Relative energy (kJ/mol) of conformers for bound PEG-drug molecules obtained from xTB calculations: the reference represents the most stable conformer, as discussed in the main text.

Drug	PEG-drug	PEG-drug 2
HINA	0	139
HINB	0	95
MAYA	0	95
CELB	0	70

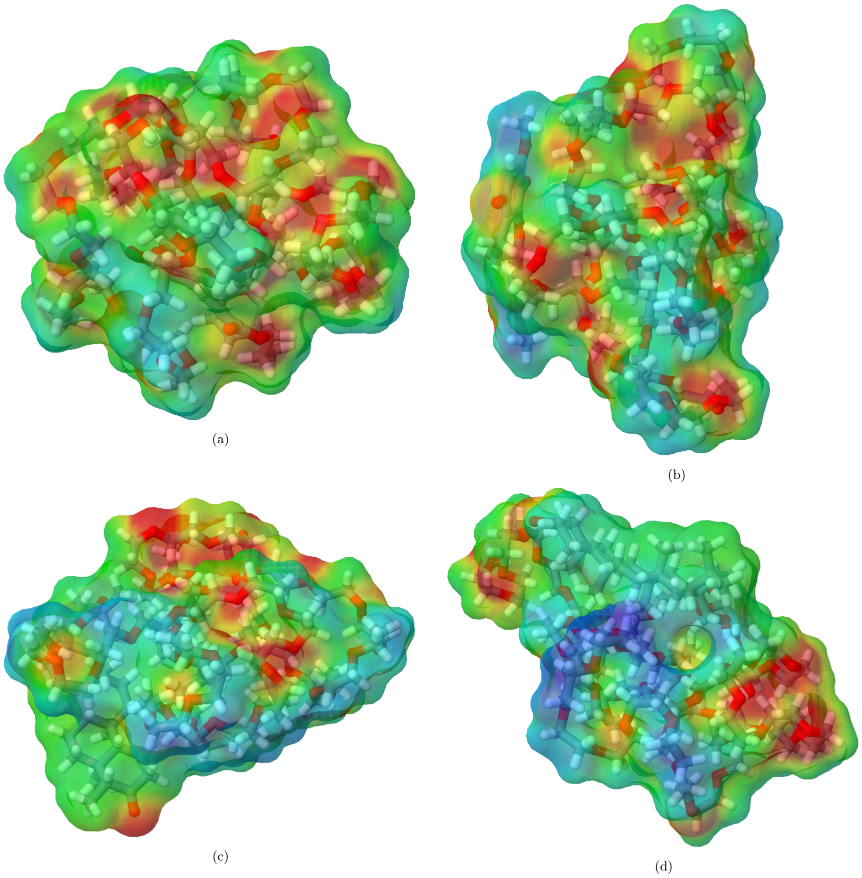

Figure S2: Visualization of Molecular Electronic Potential obtained from DFT calculations for PEG.drug complexes of HINA (a), HINB (b), MAYA (c), and CELB (d).

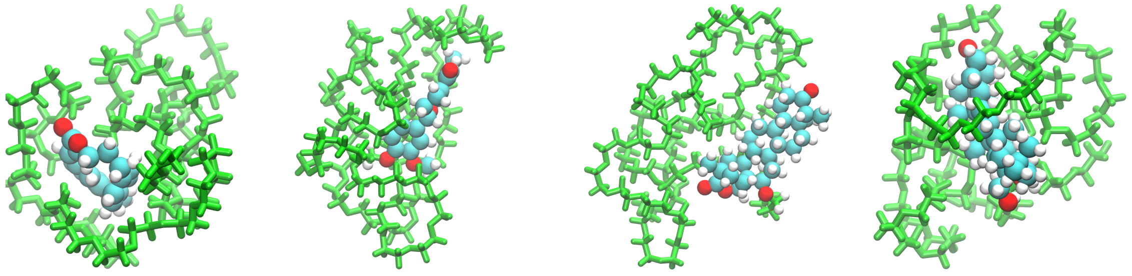

(a) (b) (c) (d)

Figure S3: Optimized structures of covalent bound PEG-drug 2 molecules obtained from xTB calculations. The PEG fragments are presented in green color for a better visualization.

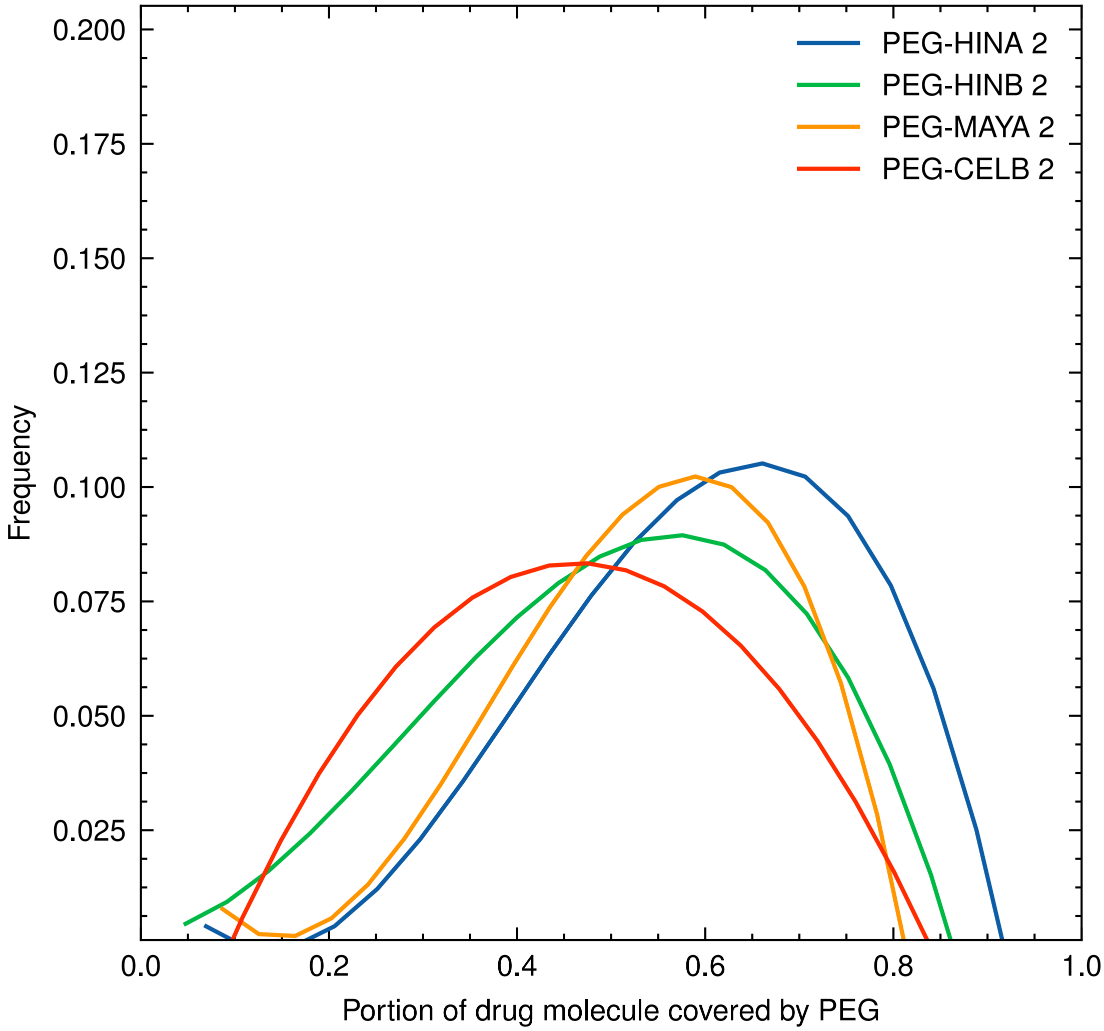

Figure S4: Histograms of the fraction of drug surface covered by PEG molecule in bound systems PEG-drug 2.
